# Complete genome analysis of a novel E3-partial-deleted human adenovirus type 7 strain isolated in Southern China

**DOI:** 10.1186/1743-422X-8-91

**Published:** 2011-03-04

**Authors:** Xiaobo Su, Xingui Tian, Qiwei Zhang, Haitao Li, Xiao Li, Huiying Sheng, Youshao Wang, Houbo Wu, Rong Zhou

**Affiliations:** 1 Key Laboratory of Tropical Marine Environmental Dynamics, South China Sea Institute of Oceanology, Chinese Academy of Sciences, Guangzhou 510301, PR China; 2 State Key Lab of Respiratory Disease, the First Affiliated Hospital of Guangzhou Medical College, Guangzhou Medical University, Guangzhou 510120, PR China; 3 Department of Microbiology, School of Public Health and Tropical Medicine, Southern Medical University, Guangzhou 510515, PR China; 4 Guangzhou Woman and Children's Medical Center, Guangzhou 510120, PR China; 5 Graduate University of Chinese Academy of Sciences, Beijing 100049, PR China

## Abstract

Human adenovirus (HAdV) is a causative agent of acute respiratory disease, which is prevalent throughout the world. Recently there are some reports which found that the HAdV-3 and HAdV-5 genomes were very stable across 50 years of time and space. But more and more recombinant genomes have been identified in emergent HAdV pathogens and it is a pathway for the molecular evolution of types. In our paper, we found a HAdV-7 GZ07 strain isolated from a child with acute respiratory disease, whose genome was E3-partial deleted. The whole genome was 32442 bp with 2864 bp deleted in E3 region and was annotated in detail (GenBank: HQ659699). The growth character was the same as that of another HAdV-7 wild strain which had no gene deletion. By comparison with E3 regions of the other HAdV-B, we found that only left-end two proteins were remained: 12.1 kDa glycoprotein and 16.1 kDa protein. E3 MHC class I antigen-binding glycoprotein, hypothetical 20.6 kDa protein, 20.6 kDa protein, 7.7 kDa protein., 10.3 kDa protein, 14.9 kDa protein and E3 14.7 kDa protein were all missing. It is the first report about E3 deletion in human adenovirus, which suggests that E3 region is also a possible recombination region in adenovirus molecular evolution.

## Introduction

Human adenoviruses (HAdVs) are implicated in a wide range of human diseases, including respiratory, ocular, metabolic, renal and gastrointestinal. They are responsible for 5-10% of lower respiratory tract infections in infants and children throughout the world. HAdV-7, a member of the B1 subspecies, causes acute respiratory disease (ARD). This pathogen is identified in epidemics, is highly virulent and is associated with clinical manifestations of considerable severity including residual lung damage and fatal outcomes[1]. Previous reports suggested that HAdV-3 and -5 are very stable across 50 years of time and space [2,3], which is common in DNA viruses. But HAdV in general are known to undergo recombination. Earlier studies demonstrated in vitro recombination. But more and more isolates, which were isolated from adenovirus epidemic, undergo new recombination between adenovirus types, which leaded to new"intermediates" or subtypes[4]. All the evidence supports the hypothesis that genome recombination drives the molecular evolution of HAdV types. In our research, we found a HAdV-7 strain isolated from a child with acute respiratory disease, with a large portion of E3 region deleted. The whole genome was annotated (GenBank: HQ659699). It hints that E3 region is also important in adenovirus recombination and molecular evolution.

## Materials and methods

### 1. Cells, virus and Preparation of viral DNA

The virus strain (designated HAdV-7 GZ07) in this study was isolated from nasal aspirates of a child with ARD in southern China in 2007. The Nasal aspirate specimen was inoculated to HEp-2 cells for isolation, which was maintained in minimal essential medium supplemented with 100 IU penicillin ml^-1^, 100 μg streptomycin ml^-1 ^and 2% (v/v) fetal bovine serum. The cells were observed for 1-2 weeks for CPE, and the supernatant was identified by a neutralization assay with type-specific reference antisera raised in rabbits by conventional procedures. Type-specific primers designed to the hypervariable regions (HVRs) of the HAdV hexon were also utilized to correctly identify the serotypes. Viral DNA was extracted by using a previously described method [5,6].

### 2. DNA restriction analysis

Restriction analysis was performed using restriction endonucleases (*Bam*HI, *Eco*RI, *Eco*RV, *Hind*III, *Sal*I, *Sma*I) and the restriction profiles were compared with those of prototype and other genome-types described in the literature and the genome-type denomination system [7].

### 3. DNA sequencing and analysis

According to the published sequences of HAdV-7 and others types, the PCR primer pairs were designed to amplify the fragments of the HAdV-GZ09 by using the isolated viral DNA. These fragments were either cloned and sequenced subsequently or sequenced directly from the amplicon. It was sequenced by primer walking with overlapping sequencing reactions. For confirmation of the exact ends of the ITR sequence, a method described by Zhang [5] was followed. All of the reported sequences are the result of at least three sequencing reactions. The sequencing reactions were carried out by using an ABI Prism BigDye Terminator v3.1 Cycle Sequencing Ready Reaction kit with Ampli*Taq *DNA polymerase on an ABI 3730 DNA sequencer (Applied Biosystems). Unresolved and ambiguous sequences were resequenced with primers close to the regions in question.

Sequence assembly was carried out with the program SeqMan 5.00 from the DNASTAR software package. The genome sequence of HAdV-7 GZ07 was firstly blasted in Genebank using megablast program, then annotated by parsing the 32442 bases into 1-kb non-overlapping segments which were queried systematically against the nonredundant NCBI database using the BLASTX program [8]. Default parameters of word size = 3 and expectation = 10, with the BLOSUM62 substitution matrix and with gap penalties of 11 (existence) and 1 (extension), were applied to these analyses. Low complexity sequences were filtered out of the queries, as per the BLAST algorithm. Genome annotation, analysis of non coding DNA motifs and functional protein motifs were performed by using the web based gene prediction software GENEMARK software [9] and determined putative proteins were performed with blastp from NCBI http://www.ncbi.nlm.nih.gov/BLAST/.

Whole-genome alignment and comparisons of the sequences from HAdVs were performed by using the dot-plot software Advanced PipMaker http://pipmaker.bx.psu.edu/cgi-bin/pipmaker?advanced, which aligns long genomic DNA sequences quickly and with good sensitivity [10].

E3 region of HAdV-7 GZ07 strain was analyzed and compared with that of the other HAdV-B strains.

CLUSTALX was used to perform multiple-sequence alignments of adenovirus E3 sequences. Phylogenetic analysis was performed with the MEGA software package (version 4.1). The phylogenetic trees were constructed with the neighbor-joining method. Bootstrap analysis was performed with 1,000 pseudoreplicates.

## Results

### 1. Confirmation of serotype and genome type

**Figure 1:**
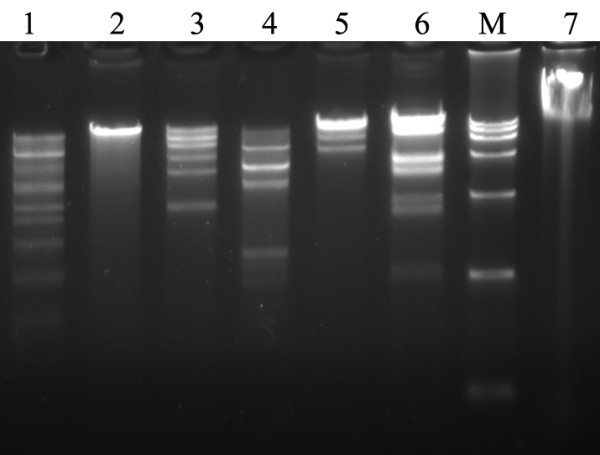
**Restriction endonuclease digestion of HAdV7-gz07 virus genome**. Products produced by restriction enzyme digestion of the genome of HAdV7-gz07. 1: *Bam*HI; 2: *Eco*RI; 3: *Eco*RV; 4:*Hind*III; 5: SalI; 6: *Sma*I; 7: genome without digestion; M: DNA marker DL15000.

Typical CPE was found in cells inoculated with HAdV-7 GZ07 strain and virus could be neutralized specifically by mice serum against HAdV-7. Type-specific PCR assay also indicated that this strain was serotype 7. Further genome-typing results of restriction profiles found difference between this strain and Gomen strain (Figure 1).

### 2. General properties of the HAdV7-GZ07 genome sequence

The complete genome of HAdV7-GZ07 is 32442 bp in length with a base composition of 26.1% G, 26.0% C, 23.1% A and 24.8% T. The G+C content is 52.1%, which is similar to that of other members of HAdV-B (50-52%) (Shenk, 2001). We identified 41 coding regions that are homologue to previously described gene products of other human adenoviruses. The annotation of the predicted coding gene regions is listed in Additional file 1: Table S1 (GenBank: HQ659699).

### 3. Whole genome comparison and E3 region analysis

**Figure 2:**
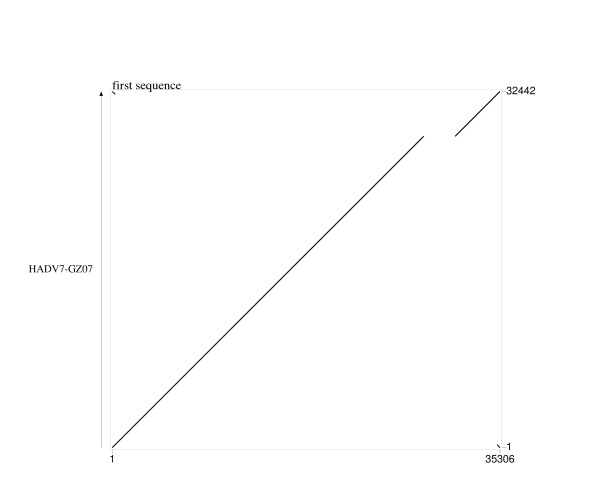
**Whole-genome analyses of the HAdV-7 strain GZ07 and Gomen sequences**.

HAdV-7 Gomen strain genome was chose as a reference strain for whole genome comparison [11]. PipMaker analysis suggested that there is an obvious genome deletion in HAdV7-GZ07 strain (Figure 2). Under detailed scrutiny, 2864 bp was deleted in E3 region between nt 28365-31228; only left-end two proteins were remained, 12.1 kDa glycoprotein and 16.1 kDa protein. E3 MHC class I antigen-binding glycoprotein, hypothetical 20.6 kDa protein, 20.6 kDa protein, 7.7 kDa protein., 10.3 kDa protein, 14.9 kDa protein and E3 14.7 kDa protein were all missing. The other parts shared close identity with respect to nucleotide sequences, with only two gaps and 14 bases mutation compared with Gomen strain. (Additional file 2: Table S2)

### 4. E3 region phylogenetic analysis

**Figure 3:**
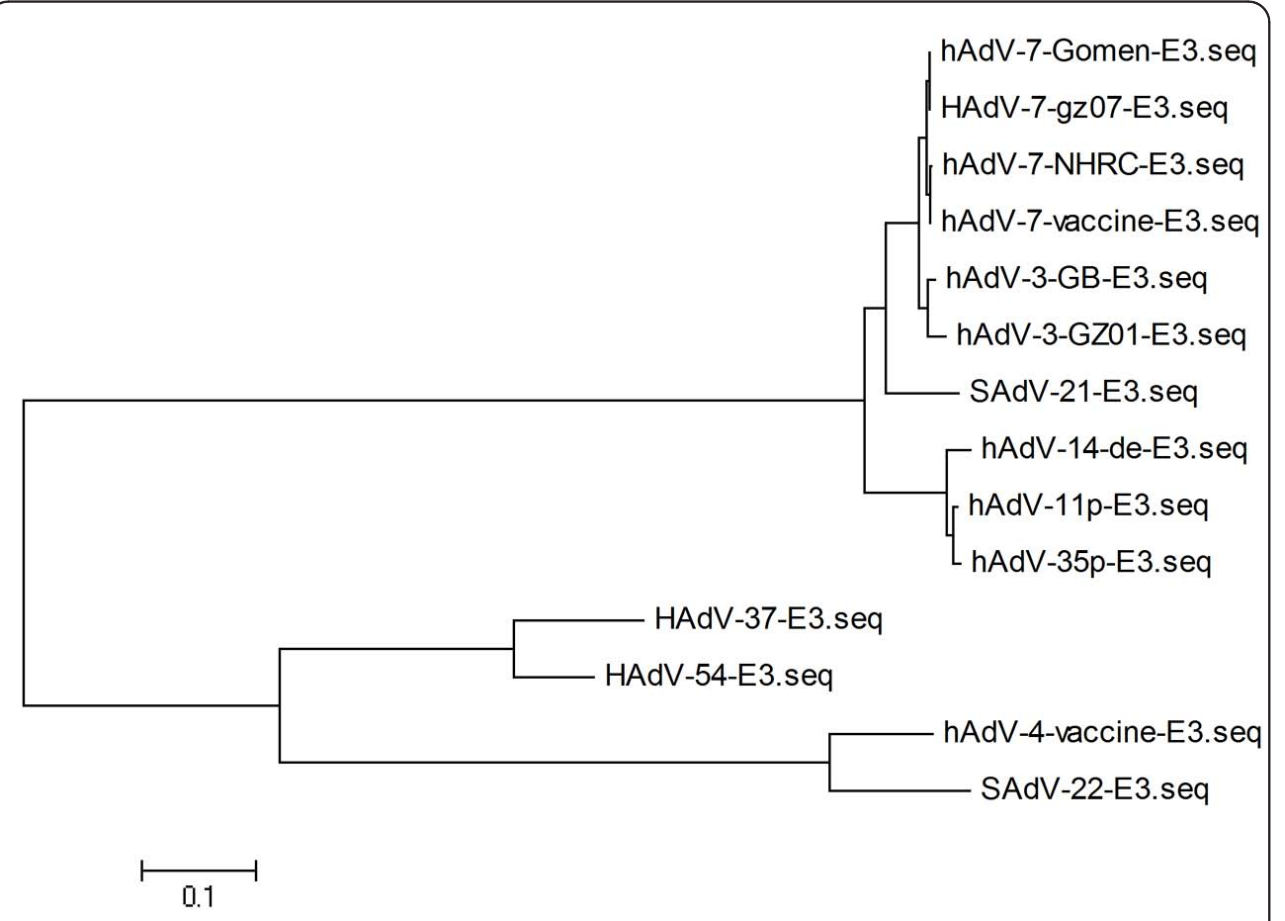
**Phylogenetic analysis of E3 regions of selective HAdV subtypes**. The GenBank accession numbers of the viruses used are: HAdV7-Gomen (AY594255), HAdV7-NHRC1315 (AY601634.1), HAdV7-vaccine (AY594256.1), HAdV3-GZ01 (DQ099432.4), HAdV3-GB (AY599834.1), SAdV-21 (AC_000010), HAdV11 (AY598970.1), HAdV14 (AY803294.1), HAdV35 (AY271307.1), HAdV4 (AY594254), SAdV-22 (AY530876), HAdV54 (AB448770.2), HAdV37 (DQ900900.1).

A phylogenetic tree was constructed based on the multiple alignments of the E3 region sequence data using the program MEGA 4.1 by the neighbor-joining method (Figure 3). The tree shows the phylogenetic relationship among the selective adenovirus isolates. As can be seen HAdV-7-GZ07 strain is very closed with Gomen strain.

## Discussion

Although E3 is non-essential for viral replication in vitro, experiments with both mice and cotton rats have shown that it does play an important role in pathogenesis[12,13]. The size and composition of the E3 transcription unit vary considerably among Ad species. The E3 region within adenovirus genomes encodes proteins that modulate the host immune response to infection and are not essential for viral growth in vitro [14]. The HAdV-7 E3 region was found to encode the 12.1-, 16.1-, 19.3-, 7.7-, 10.3-, 14.9-, and 14.7-kDa proteins. Additionally, two different 20.6-kDa proteins were contained within this transcript [11]. The 12.1-kDa protein has significant identity to an immunomodulating E3 protein in HAdV-7 Gomen strain. A glycoprotein of 16.1-kDa has homologs in other HAdV species. The 19.3-kDa protein is a major histocompatibility class I antigen-binding glycoprotein that prevents the lysis of adeno-infected host cells by cytotoxic T-lymphocytes [15]. Both 20.6-kDa proteins are similar to the CR1 (conserved region 1)-containing proteins in the E3 region of other HAdVs and SAdVs. CR1 alpha and beta were described as species HAdV-A specific gene products[16]. Prediction of transmembrane domains suggested that both gene products were type Ia transmembrane proteins. The 7.7-kDa protein is reported to insert itself into the host cell membrane; its function is yet to be determined. The E3 7.7 K ORF appears to be another area of the Ad genome in which genetic diversity may be generated by illegitimate recombination[17]. A HAdV E3 transmembrane protein has identity to the HAdV-7 10.3-kDa protein. This may have a role in downregulating the epidermal growth factor (EGF) receptor [15]. The known RID (receptor internalization and degradation) alpha and beta proteins are present in the E3 transcription units of all HAdV. Both proteins are non-covalently associated integral membrane proteins (YxxO motifs function as signals for transport and internalization into lysosomes/endosomes RID alpha is a hydrophobic protein and appears in two isoforms[16]). The last two ORFs of the E3 region encodes 14.9-and 14.7-kDa proteins that are present in all species of HAdV species[18]. It has been shown to be located in the cytosol and nucleolus, functioning as an inhibitor of TNF mediated cell lysis [19].

Homologous recombination has been recognized as an important mechanism of evolution of adenovirus genomes [20]. In some types, e.g. HAdV-3 and HAdV-5, the genomes are very stable [2,3,21]. But more and more reports found new recombination between adenovirus subtypes, which leaded to new types [22]. Illegitimate recombination has previously been proposed to contribute to Ad evolution by driving hexon sequence variation and serotype differentiation [23,24]. Hypervariability in the hexon gene among Ad serotypes can be explained as a response to host-immune pressure [25]. The detail mechanism in adenovirus recombination was not known. Some species or types may be amenable to recombination based on sequence, e.g., hotspots, and biology, e.g., cell tropism and coinfection [21]. Human recombinase proteins may also have a propensity to bind certain sequences in adenovirus genomes.

## Competing interests

The authors declare that they have no competing interests.

## Authors' contributions

XS and XT contributed in the study design and obtaining PCR data and genome sequencing. QZ gave a critical view of manuscript writing. HL performed partial molecular biology assays. HS collected the clinical samples. XL and YW participated in data analysis. HW and RZ was responsible for the planning of the study, data analysis. All authors have read and approved the final manuscript.

## Supplementary Material

Additional file 1 **Table S1. HAdV7-GZ07 strain genome-sequence annotation**. DNA sequence motifs and Forty-three coding regions are identified and located on the HAdV7-gz07 genome sequence. The hypothetical and predicted proteins are marked as 'Hypo.'. Nucleotide positions of the start/stop codons and of the applicable splice sites are noted in the 5' to 3' direction. Functionality, which is embedded within the complementary strand is designated by 'c'. Click here for file

Additional file 2 **Table S2. E3 region comparison of HAdV-B subtypes**. Click here for file

## References

[B1] Dudding BA Wagner SC Zeller JA Gmelich JT French GR Top FH Jr Fatal pneumonia associated with adenovirus type 7 in three military trainees N Engl J Med 1972 286 1289 1292 10.1056/NEJM197206152862403 4337012

[B2] Seto J Walsh MP Metzgar D Seto D Computational analysis of adenovirus serotype 5 (HAdV-C5) from an HAdV coinfection shows genome stability after 45 years of circulation Virology 2010 404 180 186 10.1016/j.virol.2010.05.010 20627349

[B3] Mahadevan P Seto J Tibbetts C Seto D Natural variants of human adenovirus type 3 provide evidence for relative genome stability across time and geographic space Virology 2010 397 113 118 10.1016/j.virol.2009.10.052 19932910

[B4] Walsh MP Seto J Jones MS Chodosh J Xu W Seto D Computational analysis identifies human adenovirus type 55 as a re-emergent acute respiratory disease pathogen J Clin Microbiol 2010 48 991 993 10.1128/JCM.01694-09 20042633 PMC2832463

[B5] Zhang Q Su X Gong S Zeng Q Zhu B Wu Z Peng T Zhang C Zhou R Comparative genomic analysis of two strains of human adenovirus type 3 isolated from children with acute respiratory infection in southern China J Gen Virol 2006 87 1531 1541 10.1099/vir.0.81515-0 16690917

[B6] Zhang Q Su X Seto D Zheng B-j Tian X Sheng H Li H Wang Y Zhou R Construction and characterization of a replication-competent human adenovirus type 3-based vector as a live-vaccine candidate and a viral delivery vector Vaccine 2009 27 1145 1153 10.1016/j.vaccine.2008.12.039 19146906

[B7] Li QG Wadell G Analysis of 15 different genome types of adenovirus type 7 isolated on five continents J Virol 1986 60 331 335 3018298 10.1128/jvi.60.1.331-335.1986 PMC253937

[B8] Altschul SF Madden TL Schaffer AA Zhang J Zhang Z Miller W Lipman DJ Gapped BLAST and PSI-BLAST: a new generation of protein database search programs Nucleic Acids Res 1997 25 3389 3402 10.1093/nar/25.17.3389 9254694 PMC146917

[B9] Besemer J Borodovsky M Heuristic approach to deriving models for gene finding Nucleic Acids Res 1999 27 3911 3920 10.1093/nar/27.19.3911 10481031 PMC148655

[B10] Schwartz S Zhang Z Frazer KA Smit A Riemer C Bouck J Gibbs R Hardison R Miller W PipMaker--a web server for aligning two genomic DNA sequences Genome Res 2000 10 577 586 10.1101/gr.10.4.577 10779500 PMC310868

[B11] Purkayastha A Su J Carlisle S Tibbetts C Seto D Genomic and bioinformatics analysis of HAdV-7, a human adenovirus of species B1 that causes acute respiratory disease: implications for vector development in human gene therapy Virology 2005 332 114 129 10.1016/j.virol.2004.10.041 15661145

[B12] Sharma S Andersson A Adenovirus E3 proteins help tumors to evade innate and adaptive immune responses Cancer Biol Ther 2009 8 1133 1135 10.4161/cbt.8.12.8616 19458480

[B13] Bortolanza S Bunuales M Alzuguren P Lamas O Aldabe R Prieto J Hernandez-Alcoceba R Deletion of the E3-6.7K/gp19K region reduces the persistence of wild-type adenovirus in a permissive tumor model in Syrian hamsters Cancer Gene Ther 2009 16 703 712 10.1038/cgt.2009.12 19229289

[B14] Windheim M Hilgendorf A Burgert HG Immune evasion by adenovirus E3 proteins: exploitation of intracellular trafficking pathways Curr Top Microbiol Immunol 2004 273 29 85 14674598 10.1007/978-3-662-05599-1_2

[B15] Wold WS Gooding LR Region E3 of adenovirus: a cassette of genes involved in host immunosurveillance and virus-cell interactions Virology 1991 184 1 8 10.1016/0042-6822(91)90815-S 1831308

[B16] Burgert HG Blusch JH Immunomodulatory functions encoded by the E3 transcription unit of adenoviruses Virus Genes 2000 21 13 25 10.1023/A:1008135928310 11022786

[B17] Kajon AE Xu W Erdman DD Sequence polymorphism in the E3 7.7K ORF of subspecies B1 human adenoviruses Virus Res 2005 107 11 19 10.1016/j.virusres.2004.06.005 15567028

[B18] Davison AJ Benko M Harrach B Genetic content and evolution of adenoviruses J Gen Virol 2003 84 2895 2908 10.1099/vir.0.19497-0 14573794

[B19] Horton TM Tollefson AE Wold WS Gooding LR A protein serologically and functionally related to the group C E3 14,700-kilodalton protein is found in multiple adenovirus serotypes J Virol 1990 64 1250 1255 2304142 10.1128/jvi.64.3.1250-1255.1990 PMC249240

[B20] Sambrook J Sleigh M Engler JA Broker TR The evolution of the adenoviral genome Ann N Y Acad Sci 1980 354 426 452 10.1111/j.1749-6632.1980.tb27983.x 7013621

[B21] Seto J Walsh M Mahadevan P Zhang Q Seto D Applying Genomic and Bioinformatic Resources to Human Adenovirus Genomes for Use in Vaccine Development and for Applications in Vector Development for Gene Delivery Viruses 2010 2 1 26 10.3390/v2010001 PMC3185558 21994597

[B22] Walsh MP Seto J Jones MS Chodosh J Xu W Seto D Computational analysis identifies human adenovirus type 55 as a re-emergent acute respiratory disease pathogen J Clin Microbiol 2010 48 991 993 10.1128/JCM.01694-09 20042633 PMC2832463

[B23] Crawford-Miksza LK Schnurr DP Adenovirus serotype evolution is driven by illegitimate recombination in the hypervariable regions of the hexon protein Virology 1996 224 357 367 10.1006/viro.1996.0543 8874497

[B24] Rebelo-de-Andrade H Pereira C Giria M Prudencio E Brito MJ Cale E Taveira N Outbreak of acute respiratory infection among infants in Lisbon, Portugal, caused by human adenovirus serotype 3 and a new 7/3 recombinant strain J Clin Microbiol 2010 48 1391 1396 10.1128/JCM.02019-09 20147640 PMC2849616

[B25] Crawford-Miksza LK Nang RN Schnurr DP Strain variation in adenovirus serotypes 4 and 7a causing acute respiratory disease J Clin Microbiol 1999 37 1107 1112 10074533 10.1128/jcm.37.4.1107-1112.1999 PMC88656

